# Measuring activation during behavioral activation therapy: a proof-of-concept study using smartphone sensors and LLM-derived ratings in adolescents with anhedonia

**DOI:** 10.1038/s44277-025-00045-w

**Published:** 2025-10-13

**Authors:** Hadar Fisher, Nigel M. Jaffe, Habiballah Rahimi-Eichi, Erika E. Forbes, Diego A. Pizzagalli, Justin T. Baker, Christian A. Webb

**Affiliations:** 1 McLean Hospital, Belmont, MA USA; 2 Department of Psychiatry, Harvard Medical School, Boston, MA USA; 3 Department of Psychiatry, University of Pittsburgh, Pittsburgh, PA USA; 4 Noel Drury, M.D. Institute for Translational Depression Discoveries, University of California, Irvine, CA USA

**Keywords:** Therapeutics, Outcomes research

## Abstract

Adolescent depression remains a major public health concern, and Behavioral Activation (BA), a brief therapeutic intervention designed to reduce depression-related avoidance and boost engagement in rewarding activities, has shown encouraging results. Still, few studies directly measure the hypothesized mechanism of “activation” in daily life, especially using low-burden, ecologically valid methods. This proof-of-concept study evaluates the validity of two technology-based approaches to measuring activation in adolescents receiving BA: smartphone-based mobility sensing and large language model (LLM) ratings of free-response text. Adolescents (*n* = 38, ages 13–18) receiving 12-week BA therapy for anhedonia completed daily ecological momentary assessment (EMA) reporting on positive and negative affect. GPT-4o was used to rate behavioral activation from EMA free-text entries. A subsample (*n* = 13) contributed passive smartphone sensing data (e.g., accelerometer activity, GPS-derived mobility). Activation and symptoms were assessed weekly via self-report. GPT-derived activation ratings correlated positively with passive sensing indicators (number of places visited, time away from home) and self-reported activation. Within-person increases in GPT-rated activation were associated with higher daily positive affect and lower negative affect. Passive sensing features also forecasted weekly improvements in anhedonia and depressive symptoms. Associations emerged primarily at the within-person level, suggesting that changes in activation relative to one’s own baseline are clinically meaningful. This study demonstrates the feasibility and validity of passively measuring behavioral activation in adolescents’ daily lives using smartphone data and LLMs. These tools hold promise for advancing data-informed psychotherapy by tracking therapeutic processes in real time, reducing reliance on self-report, and enabling personalized, adaptive interventions. Clinical Trial Registry: NCT02498925.

## Lay summary

Behavioral Activation therapy helps depressed teens by encouraging them to engage in more rewarding activities. We tested whether smartphones and LLM could automatically track this 'activation' in daily life. Using movement data from phones and LLM-based ratings from 38 teens in therapy, we found these digital tools accurately detected when teens were more active and higher activation predicted better mood and fewer depression symptoms. This technology could help therapists monitor progress in real-time and personalize treatment more effectively.

## Introduction

Rates of depression increase significantly during adolescence, marking this as a critical developmental period for intervention [1]. Yet, even first-line treatments, such as cognitive behavioral therapy, are not consistently effective for youth [2]. Anhedonia, or loss of interest and pleasure, is a core feature of depression that predicts poorer course of illness and worse treatment outcomes [3]. Treatments that target anhedonia may lead to reductions in other depressive symptoms [4].

Behavioral Activation (BA) therapy aims to reduce anhedonia by targeting patterns of avoidance and withdrawal and increasing engagement with rewarding activities [5]. Given its relative simplicity and clear focus on behavioral reinforcement of rewards, BA may be especially well-suited to efficiently target adolescent anhedonia, and promising evidence exists in youth samples [6, 7]. A core assumption of BA is that increased “activation”(in this article, we use “Behavioral Activation” or “BA” to refer to the manualized treatment approach described by [5] and [6]; by contrast, we use “activation” to refer to the *construct* of behavioral activation, i.e., the putative mechanism through which patients become activated in this treatment. See [8] for a review of how this construct is often measured) in daily life leads to symptom change [9], but few studies have rigorously measured whether this is the case [8]. To assess activation most of these studies rely on self-report questionnaires, which are limited by recall bias, social desirability, and participant burden [8]. There is a pressing need for objective, low-burden, and ecologically valid methods to track activation in daily life. Tools that assess activation *between* sessions could help therapists monitor treatment progress and make timely adjustments, consistent with calls for data-informed psychotherapy [10].

Passive sensing through smartphones offers a low-burden, continuous, and objective way to assess real-world behavior [11]. Mobility-related passive sensing features are particularly relevant for assessing behavioral proxies for activation, such as activity levels (accelerometer) or percent of time spent outside the home (GPS). Growing evidence suggests smartphone sensors can be used to predict daily and even hourly fluctuations in depressed mood [12], including in youth [13–15]. In a BA study [16], demonstrated congruence between questionnaire-assessed activation and several smartphone metrics, including step count, time spent at home, time in conversation, and screen unlocks, albeit in a very small sample (3 case studies) that requires replication. Despite substantial advantages, passive sensing measures alone lack qualitative context and therefore provide limited insight into the emotional or motivational aspects of behavior.

Unobtrusive language samples offer another key source of information with regard to both qualitative context and quantitative features (e.g., sentiment) that can be used in conjunction with passive sensing to estimate or forecast emotional states and behavior. For example, promising results have been obtained by applying Linguistic Inquiry and Word Count (LIWC) to measure activation in online therapy chat logs [17] obtained promising results by applying Linguistic Inquiry and Word Count (LIWC) to measure activation in online therapy chat logs. However, LIWC required a lexicon of activation-relevant words from their specific dataset, limiting generalizability. In contrast, large language models (LLMs) have been shown to accurately identify psychological constructs in text, while requiring no sample-specific training data [18]. Recent work has demonstrated the potential of LLMs to estimate emotion from daily free response text [19]. Additionally, frequent self-reports of daily activities provide ideal material for LLMs to rate activation, with less risk of typical self-report biases.

This proof-of-concept study aimed to evaluate the validity and utility of two novel, technology-based measures of activation: LLM-derived ratings of daily text entries and smartphone-based passive sensing of mobility patterns. These measures were examined in relation to daily positive and negative emotion, as well as weekly anhedonia, depressive symptoms, and a traditional self-report measure of activation. We hypothesized that the LLM-derived and passive sensing indicators of activation would be positively associated with each other and with self-reported activation, supporting their convergent validity. Furthermore, we hypothesized that these measures would also demonstrate *criterion* validity, such that higher activation as captured by these methods would be associated with increased daily positive emotion, decreased daily negative emotion, and greater weekly improvement in anhedonia and depression symptoms.

## Materials and methods

### Participants and procedure

Participants were 38 adolescents recruited from the greater Boston area for a BA treatment trial for adolescent anhedonia from January 2016 to November 2021. See supplement (Table S1) for demographic and clinical characteristics. For more information on study design and inclusion/exclusion criteria, see [7]. Approval was obtained from the Mass General Brigham IRB, and all participants gave informed consent and/or assent.

Participants received 12 weekly, 60-minute, individual therapy sessions, per the [6] BA manual. Before each session participants were asked to rate their anhedonia, using the 14-item Snaith‐Hamilton Pleasure Scale (SHAPS; [20, 21]), depression, using the 20-item Center for Epidemiological Studies Depression scale (CES-D; [22]), and activation, using the 9-item Behavioral Activation for Depression Scale - Short Form (BADS – SF; [23]). (See Supplement Section S1 for more details about the weekly self-report measures.) During the treatment, every other week participants completed a 5-day (2-3 surveys per day) bursts of ecological momentary assessments (EMA) using the MetricWire app. At each prompt, participants rated their positive affect (PA) and negative affect (NA) by responding to 6 items, each anchored by a 5-point Likert scale. Next, they were asked to provide free-text responses about what they were doing right before, with whom they were interacting, and the most enjoyable and stressful events since the previous prompt. (See Supplement Section S1, https://clinicaltrials.gov/study/NCT02498925 and [4] for further details about the EMA items and sampling protocol). Passive sensor data were collected continuously using the Beiwe [24] smartphone platform for a subset of participants (*n* = 13), as this data collection method was added to the protocol after initial participant enrollment had begun.

### Feature derivation

EMA-derived PA was calculated as the mean score of “happy,” “interested,” and “excited” items; NA was the mean of “sad,” “nervous,” and “angry” items. Typed free-text responses provided by participants as part of their EMA surveys (see Section S2 for the specific open questions) were analyzed using OpenAI’s GPT-4o model, accessed via the Python *openai* package. Using the API ensured application of the same standardized prompt, fixed model parameters (e.g., temperature), and consistent, reproducible outputs across the dataset. The standardized prompt included the clinical definition of behavioral activation (Dimidjian et al., 2011), rating criteria (1 = least active/passive/avoidant to 5 = very high activity with strong reinforcement of pleasure, mastery, or problem-solving), and guidelines for evaluating current activities, social interactions, enjoyable events, and responses to stressful situations (see Supplement Section S2 for the full prompt). The model was instructed to output a single numeric rating [1–5] along with a rationale, based on explicit rating criteria (e.g., passivity vs. activity, social engagement, mastery/enjoyment, and problem-solving vs. avoidance). See Table S8 for examples of participant text with corresponding GPT ratings and explanations. Participant responses were manually deidentified prior to analysis by removing or replacing personal identifiers (e.g., names, places). To evaluate validity, a trained human rater applied the same prompt and criteria to a random 25% subsample (n = 478) of EMA responses. Human and GPT-4o ratings demonstrated substantial agreement (weighted Cohen’s κ = 0.77, 95% CI [0.74, 0.81]), supporting the validity of the automated rating.

Passive sensor features were extracted from participants’ smartphone data to characterize behavioral patterns linked to mood and functioning using the publicly available DPlocate and DPphone software packages [25]. Derived measures included: an hourly activity score (*ActScore*), derived from aggregated accelerometer data to reflect physical activity intensity throughout the day; daily percent home (*percentHome*), indicating the proportion of time spent at the inferred home location based on nighttime GPS clustering; daily distance from home (*homDist*), computed as the mean Euclidean distance between GPS samples and the home location; daily mobility area (*radiusMobility*), representing the spatial dispersion of movement via radius of gyration; and places visited daily (*DayPlaces*), estimated using spatiotemporal clustering to identify distinct locations visited each day. These features were selected based on prior evidence linking mobility, location stability, and physical activity to affective states. (See Supplement Section S3 for detailed definitions of each passive sensor variable.)

### Analytic strategy

First, we computed daily means for the hourly passive sensing variables, EMA affect variables, and GPT ratings. The daily variables were used to examine associations with NA and PA at the *daily* level. For *week*-level analyses, we predicted BADS, SHAPS, and CES-D scores using weekly means of the GPT ratings and passive sensing variables. For all analyses, we used multilevel modeling (MLM) to estimate both within-person (person-mean centered) as well as between-person (participant mean) effects. Models included random intercepts for participants and were conducted using the lme4 package in R. To assess convergent validity, we examined associations between GPT ratings, passive sensing variables, and BADS scores. (We also conducted repeated measures correlation (rmcorr) analyses. Results from rmcorr were nearly identical to those obtained from multilevel models; therefore, we report them in the supplement only (see Supplementary Figure S1 to S4)). To assess criterion validity, we tested whether higher GPT and passive sensor-derived activation were associated with more positive and less negative daily affect, controlling for prior-day affect, and with lower weekly symptoms of anhedonia and depression, controlling for prior-week scores. Models were fit using restricted maximum likelihood (REML), which provides unbiased variance component estimates in multilevel models, especially with small or unbalanced samples. Missing data were handled automatically through REML estimation, which uses all available observations under the missing at random assumption, allowing participants to contribute data even when some time points are missing [26, 27]. Comprehensive evaluations of missing data mechanisms are reported in the Supplement Section S4. No a priori sample size calculation was conducted, as this was a secondary proof-of-concept analysis using data from an existing treatment trial. The analytic sample consisted of all participants who completed daily EMA (n = 38) and a subset who contributed passive smartphone sensing data (n = 13). All analysis code is publicly available at https://osf.io/jhczf/files/osfstorage.

## Results

Thirty-eight participants completed 50.24 EMA surveys on average (*SD* = 27.52) across the entire 12-week treatment period. EMA compliance decreased over the course of treatment, from 62% in in the first two weeks to 52% in the last two weeks (Table S2). For the 13 subjects with passive sensor data, a total of 46,056 hourly observations were collected (*n* = 1919 days of data total; *M* = 147.62 days; *SD* = 99.82 days). Descriptive statistics for daily assessment of smartphone passive sensing features and GPT-derived ratings are presented in the supplement (Table S3).

### Convergent validity: associations between activation indicators

#### Passive sensors and GPT-derived ratings

On days with higher-than-usual smartphone-derived activity scores (*Est*. = 0.178, *p* = 0.024) and more places visited daily (*Est*. = 0.045, *p* = 0.021), participants tended to have higher GPT-rated activation, while spending more time at home predicted lower GPT-rated activation that day (*Est*. = −0.007, *p* = 0.012). No significant between-person associations were found (see Table 1 and Figure S1).

**Table 1:** Multilevel Model Results of Passive Sensing Variables Predicting Daily GPT-Derived Activation.

Model	Predictor	N	Estimate (SE)	statistic	*p* value	95% CI
ActScore	(Intercept)	323	0.167 (4.172)	0.040	0.969	[−9.411, 9.745]
	ActScore _person-centered_	323	**0.178** (**0.078)**	**2.27**	**0.024**	**[0.024, 0.332]**
	ActScore _mean_	323	0.780 (1.16)	0.672	0.519	[−1.880, 3.440]
homDist	(Intercept)	319	2.91 (0.064)	45.39	<0.001	[2.76, 3.05]
	homDist _person-centered_	319	0.000 (0.000)	0.335	0.738	[−0.000, 0.000]
	homDist _mean_	319	0.000 (0.000)	1.95	0.075	[−0.000, 0.000]
radiusMobility	(Intercept)	319	3.021 (0.136)	22.24	<0.001	[2.722, 3.321]
	radiusMobility _person-centered_	319	0.001 (0.001)	0.924	0.356	[−0.001, 0.002]
	radiusMobility _mean_	319	−0.002 (0.005)	−0.334	0.744	[−0.013, 0.009]
percentHome	(Intercept)	319	4.388 (0.793)	5.53	<0.001	[2.651, 6.125]
	percentHome _person-centered_	319	−**0.007** (**0.003)**	−**2.53**	**0.012**	**[**−**0.012**, −**0.002]**
	percentHome _mean_	319	−0.018 (0.010)	−1.78	0.101	[−0.040, 0.004]
DayPlaces	(Intercept)	319	2.673 (0.319)	8.37	<0.001	[2.001, 3.345]
	DayPlaces _person-centered_	319	**0.045** (**0.019)**	**2.31**	**0.021**	**[0.007, 0.083]**
	DayPlaces _mean_	319	0.077 (0.078)	0.978	0.340	[−0.087, 0.240]

*ActScore* – Daily phone accelerometer activity score. *homDist* – Maximum distance (in km) from home traveled that day. *radiusMobility* – Radius (in km) covering all places visited in a day. *percentHome* – Percentage of time spent at home each day. *DayPlaces* – Number of distinct locations visited each day. Variables labeled *person-centered* reflect day-to-day fluctuations within individuals. Variables labeled *mean* reflect each participant’s average across the study period.

#### Predicting self-report activation

In weeks when participants’ GPT-rated activation was higher, their BADS scores (*Est*. = 2.05, *p* = 0.005) were also higher (see Table 2). Figure S2 shows individual trajectories of GPT-rated activation and BADS score over time. For passive sensing, at the within-person level, days with greater number of places visited and less time spent at home were associated with higher BADS scores (all *p*s < 0.01; see Table 3 and Figure S3). At the between-person level, participants who generally spent more time at home had lower BADS scores. See Tables S5 and S6 for models using the BADS activation and avoidance subscales.

**Table 2:** Multilevel Model Results of GPT-Derived Activation Ratings Predicting Weekly Self-Reported Activation and Symptom Measures.

Outcome	Term	N	Estimate (SE)	Statistic	*p* value	95% CI
Activation scale	(Intercept)	289	7.08 (6.52)	1.09	0.285	[−6.16, 20.32]
	GPT rating _person-centered_	**289**	**2.05** (**0.72)**	**2.85**	**0.005**	**[0.64 – 3.47]**
	GPT rating _mean_	289	2.81 (2.20)	1.28	0.210	[−1.66, 7.29]
Anhedonia (SHAPS)	(Intercept)	252	38.62 (7.04)	5.48	<0.001	[24.75, 52.50]
	Lag Outcome	252	0.38 (0.06)	6.31	<0.001	[0.26, 0.50]
	GPT rating _person-centered_	252	0.07 (0.46)	0.16	0.873	[−1.88, 0.68]
	GPT rating _mean_	252	−1.87 (2.38)	−0.78	0.435	[−6.56, 2.83]
Depressive Symptoms (CES-D)	(Intercept)	252	40.33 (13.57)	2.97	0.003	[13.59, 67.07]
	Lag Outcome	252	0.54 (0.05)	10.31	<0.001	[0.44, 0.64]
	GPT rating _person-centered_	252	−0.60 (0.65)	−0.93	0.355	[−1.88, 0.68]
	GPT rating _mean_	252	−4.32 (4.59)	−0.94	0.348	[−13.34; 4.73]

Variables labeled *person-centered* reflect day-to-day fluctuations within individuals. Variables labeled *mean* reflect each participant’s average across the study period.

**Table 3:** Multilevel Model Results of Passive Sensing Variables Predicting Weekly Self-Reported Activation.

Term	N	Estimate (SE)	Statistic	*p* value	95% CI
(Intercept)	147	−77.11 (69.47)	−1.11	0.293	[−231.60, 77.37]
ActScore _person-centered_	147	−3.80 (5.97)	−0.637	0.525	[−15.61, 8.01]
ActScore _mean_	147	28.92 (19.81)	1.46	0.175	[−15.14, 72.99]
(Intercept)	147	23.26 (1.99)	11.68	<0.001	[18.83, 27.69]
homDist _person-centered_	147	0.001 (0.003)	0.404	0.687	[−0.005, 0.008]
homDist _mean_	147	0.004 (0.002)	1.55	0.154	[−0.002, 0.009]
(Intercept)	147	19.59 (3.43)	5.72	<0.001	[11.93, 27.26]
radiusMobility _person-centered_	147	0.050 (0.031)	1.63	0.105	[−0.011, 0.111]
radiusMobility _mean_	147	0.366 (0.226)	1.62	0.137	[−0.139, 0.872]
(Intercept)	147	76.57 (21.01)	3.64	0.005	[29.64, 123.49]
**percentHome**_**person-centered**_	**147**	−**0.248** (**0.082)**	−**3.02**	**0.003**	**[**−**0.409**, −**0.086]**
**percentHome**_**mean**_	**147**	−**0.651** (**0.261)**	−**2.50**	**0.032**	**[**−**1.23**, −**0.069]**
(Intercept)	147	10.13 (9.05)	1.12	0.290	[−10.10, 30.36]
**DayPlaces**_**person-centered**_	**147**	**2.21** (**0.654)**	**3.38**	**0.001**	**[0.917, 3.50]**
DayPlaces _mean_	147	3.82 (2.40)	1.60	0.143	[−1.54, 9.18]

*ActScore* – Daily phone accelerometer activity score. *homDist* – Maximum distance (in km) from home traveled that day. *radiusMobility* – Radius (in km) covering all places visited in a day. *percentHome* – Percentage of time spent at home each day. *DayPlaces* – Number of distinct locations visited each day. Variables labeled *person-centered* reflect day-to-day fluctuations within individuals. Variables labeled *mean* reflect each participant’s average across the study period.

### Criterion validity: associations with daily affect and weekly symptoms

#### Predicting daily affect

Higher GPT-rated activation was associated with lower same-day negative affect (*Est*. = −0.09, *p* < 0.001) and higher positive affect (*Est*. = 0.16, *p* < 0.001, see Table 4). Among passive sensing features, only daily mobility radius significantly predicted daily positive affect at the within-person level (*Est*.= 0.002, *p* = 0.024; see Table 4 and Figure S4). (Including lagged (t-1) affect scores in the models substantially reduced the number of observations, so we chose to report results from models without lagged predictors. However, the results were consistent when controlling for lagged affect (see Supplementary Table S4)).

**Table 4:** Multilevel Model Results of *Passive Sensing Variables and* GPT-Derived Activation Predicting Daily Negative and Positive Affect.

Term	N	Negative Affect				Positive Affect			
		Estimate (SE)	Statistic	*p* value	95% CI	Estimate (SE)	Statistic	*p* value	pa_95% CI
(Intercept)	323	−17.67 (12.49)	−1.41	0.186	[−45.27, 9.92]	16.19 (7.28)	2.22	0.051	[−0.106, 32.48]
ActScore _person-centered_	323	0.100 (0.057)	1.76	0.079	[−0.012, 0.212]	0.094 (0.067)	1.39	0.164	[−0.039, 0.227]
ActScore _mean_	323	5.26 (3.47)	1.52	0.158	[−2.40, 12.92]	−4.25 (2.02)	−2.10	0.063	[−8.77, 0.271]
(Intercept)	319	1.34 (0.234)	5.74	0.000	[0.828, 1.86]	0.882 (0.153)	5.78	<0.001	[0.545, 1.22]
homDist _person-centered_	319	0.000 (0.000)	0.414	0.679	[−0.000, 0.000]	0.000 (0.000)	1.21	0.227	[−0.000, 0.001]
homDist _mean_	319	−0.000 (0.000)	−0.613	0.552	[−0.001, 0.001]	−0.000 (0.000)	−0.032	0.975	[−0.001, 0.001]
(Intercept)	319	1.67 (0.369)	4.54	0.001	[0.860, 2.49]	0.713 (0.252)	2.84	0.017	[0.157, 1.27]
radiusMobility _person-centered_	319	0.000 (0.001)	0.012	0.990	[−0.001, 0.001]	**0.002 (0.001)**	**2.27**	**0.024**	**[0.000, 0.003]**
radiusMobility _mean_	319	−0.017 (0.014)	−1.26	0.234	[−0.047, 0.013]	0.008 (0.009)	0.893	0.391	[−0.012, 0.029]
(Intercept)	319	0.862 (2.49)	0.346	0.736	[−4.62, 6.35]	3.03 (1.47)	2.07	0.064	[−0.212, 6.27]
percentHome _person-centered_	319	0.000 (0.002)	0.013	0.990	[−0.004, 0.004]	−0.000 (0.002)	−0.162	0.871	[−0.005, 0.004]
percentHome _mean_	319	0.005 (0.032)	0.172	0.866	[−0.065, 0.076]	−0.028 (0.019)	−1.47	0.171	[−0.069, 0.014]
(Intercept)	319	−0.192 (0.743)	−0.259	0.800	[−1.83, 1.44]	0.682 (0.577)	1.18	0.261	[−0.577, 1.94]
DayPlaces _person-centered_	319	−0.009 (0.014)	−0.654	0.514	[−0.037, 0.019]	0.027 (0.017)	1.62	0.107	[−0.006, 0.060]
DayPlaces _mean_	319	0.372 (0.181)	2.05	0.064	[−0.026, 0.769]	0.051 (0.140)	0.360	0.725	[−0.256, 0.357]
(Intercept)	1906	1.55 (0.901)	1.72	0.095	[−0.281, 3.38]	0.005 (0.635)	0.008	0.994	[−1.28, 1.29]
Lag affect (pos or neg)	1906	0.268 (0.022)	12.33	<0.001	[0.226, 0.311]	0.233 (0.022)	10.69	<0.001	[0.190, 0.275]
GPT-derived BA _person-centered_	**1906**	−**0.090 (0.015)**	−**6.01**	**<0.001**	**[**−**0.119**, −**0.061]**	**0.161 (0.016)**	**10.11**	**<0.001**	**[0.130, 0.192]**
GPT-derived BA _mean_	1906	−0.188 (0.306)	−0.615	0.543	[−0.810, 0.433]	0.328 (0.216)	1.52	0.137	[−0.110, 0.766]

*ActScore* – Daily phone accelerometer activity score. *homDist* – Maximum distance (in km) from home traveled that day. *radiusMobility* – Radius (in km) covering all places visited in a day. *percentHome* – Percentage of time spent at home each day. *DayPlaces* – Number of distinct locations visited each day. Variables labeled *person-centered* reflect day-to-day fluctuations within individuals. Variables labeled *mean* reflect each participant’s average across the study period.

#### Predicting weekly symptoms

At the weekly level, GPT-derived activation ratings were not significantly associated with changes in SHAPS or CES-D scores (see Table 2). For passive sensing variables, in weeks with greater number of places visited, participants had lower SHAPS (*Est*. = −0.82, *p* = 0.022) and CES-D scores (*Est*. = −2.51, *p* < 0.001). Conversely, weeks with more time spent at home were related to higher SHAPS (*Est*. = 0.15, *p* = 0.001) and CES-D scores (*Est*. = 4.47, *p* = <0.001). No between-person effects reached statistical significance (see Table 5 and Figure S5). A visual summary of the passive sensing results is presented in Table S7.

**Table 5:** Multilevel Model Results of *Passive Sensing Variables* Predicting Weekly Symptoms.

Term	N	Anhedonia				Depression			
		Estimate (SE)	Statistic	*p* value	95% CI	Estimate (SE)	Statistic	*p* value	95% CI
(Intercept)	146	48.06 (22.35)	2.15	0.102	[−15.44, 111.56]	18.19 (32.65)	0.557	0.600	[−64.46, 100.83]
Lagged Outcome	146	0.730 (0.059)	12.41	0.000	[0.610, 0.851]	0.668 (0.063)	10.53	<0.001	[0.541, 0.794]
ActScore _person-centered_	146	2.64 (3.13)	0.841	0.402	[−3.57, 8.84]	−3.64 (5.39)	−0.674	0.502	[−14.31, 7.04]
ActScore _mean_	146	−4.27 (6.37)	−0.670	0.541	[−22.39, 13.85]	3.04 (9.31)	0.327	0.757	[−20.54, 26.62]
(Intercept)	146	33.19 (0.630)	52.72	0.000	[31.33, 35.05]	28.90 (0.974)	29.65	<0.001	[26.46, 31.33]
Lagged Outcome	146	0.742 (0.059)	12.68	0.000	[0.621, 0.862]	0.658 (0.063)	10.45	<0.001	[0.533, 0.783]
homDist _person-centered_	146	0.000 (0.002)	0.228	0.820	[−0.003, 0.004]	0.000 (0.003)	0.141	0.888	[−0.005, 0.006]
homDist _mean_	146	−0.000 (0.001)	−0.570	0.607	[−0.003, 0.002]	−0.000 (0.001)	−0.089	0.933	[−0.003, 0.003]
(Intercept)	146	34.31 (1.08)	31.84	0.000	[31.24, 37.37]	31.32 (1.29)	24.20	<0.001	[27.77, 34.86]
Lagged Outcome	146	0.724 (0.059)	12.31	0.000	[0.604, 0.844]	0.656 (0.062)	10.58	<0.001	[0.533, 0.779]
radiusMobility _person-centered_	146	−0.018 (0.016)	−1.14	0.258	[−0.051, 0.014]	−0.054 (0.027)	−1.96	0.053	[−0.108, 0.001]
radiusMobility _mean_	146	−0.096 (0.071)	−1.36	0.251	[−0.300, 0.107]	−0.203 (0.086)	−2.37	0.073	[−0.436, 0.030]
(Intercept)	146	16.91 (6.29)	2.69	0.054	[−0.489, 34.32]	9.43 (9.24)	1.02	0.348	[−13.37, 32.24]
Lagged Outcome	146	0.736 (0.056)	13.13	0.000	[0.621, 0.852]	0.680 (0.060)	11.40	<0.001	[0.562, 0.799]
percentHome _person-centered_	146	**0.150** (**0.044)**	**3.44**	**0.001**	**[0.064, 0.237]**	**0.325** (**0.073)**	**4.47**	**<0.001**	**[0.181, 0.470]**
percentHome _mean_	146	0.201 (0.078)	2.57	0.061	[−0.015, 0.417]	0.241 (0.115)	2.10	0.082	[−0.042, 0.525]
(Intercept)	146	35.26 (3.03)	11.65	0.000	[26.74, 43.78]	30.74 (4.42)	6.96	0.001	[19.66, 41.82]
Lagged Outcome	146	0.718 (0.059)	12.21	0.000	[0.599, 0.837]	0.651 (0.060)	10.92	<0.001	[0.533, 0.769]
DayPlaces _person-centered_	146	−**0.819** (**0.352)**	−**2.33**	**0.022**	**[**−**1.52**, −**0.122]**	−**2.51** (**0.570)**	−**4.41**	**<0.001**	**[**−**3.64**, −**1.38]**
DayPlaces _mean_	146	−0.584 (0.802)	−0.728	0.508	[−2.84, 1.67]	−0.491 (1.17)	−0.420	0.691	[−3.43, 2.44]

*ActScore* – Daily phone accelerometer activity score. *homDist* – Maximum distance (in km) from home traveled that day. *radiusMobility* – Radius (in km) covering all places visited in a day. *percentHome* – Percentage of time spent at home each day. *DayPlaces* – Number of distinct locations visited each day. Variables labeled *person-centered* reflect day-to-day fluctuations within individuals. Variables labeled *mean* reflect each participant’s average across the study period.

## Discussion

This initial proof-of-concept study demonstrates the potential of scalable, smartphone-derived technologies to track therapeutic processes in adolescents’ daily lives. Specifically, we validated two novel technology-based measures of activation, mobility indicators from passive smartphone sensing and LLM-derived text ratings, against traditional questionnaire-rated activation. To our knowledge, this is the first study to integrate both LLM and passive sensing in adolescents’ daily lives to monitor therapeutic mechanisms.

LLM-derived activation ratings and select passive sensing indicators were positively associated with each other and with an established measure of behavioral activation (BADS), supporting their convergent validity. Notably, only some passive sensing features showed significant associations, suggesting that specific aspects of mobility may be more reliable markers of activation than others. Specifically, when adolescents visited more locations and spent less time at home, they also were found to have greater activation via self-report and LLM-derived ratings of their EMA text entries. In contrast, other mobility indicators, such as distance from home, were not associated with self-reported or LLM-derived activation, suggesting that activation is better reflected by time spent away from home and visiting *different* locations, rather than the distance traveled. These findings are consistent with prior studies linking fewer unique locations visited and more time spent at home to increased depression risk [15, 28]. However, previous research has typically assumed that mobility patterns are related to depression because they reflect activation, without directly testing whether this is the case. To that end, our results demonstrate a direct association of mobility with a therapeutic target—activation—rather than with depression symptoms alone.

The association between LLM-rated activation and BADS scores contributes to recent evidence suggesting that LLMs, such as GPT, can extract psychologically meaningful information from unstructured text [18]. Relevant to the treatment of adolescent depression, we build on emerging work showing that LLMs can be used to analyze language from psychotherapy sessions to inform clinical decision-making [29]. Importantly, our study shows that LLM-based assessments can also provide clinically relevant insights based on language generated *outside* the therapy room, offering scalable and unobtrusive ways to monitor therapeutic processes in patients’ daily lives.

These two technology-based approaches demonstrated distinct patterns in their associations with emotional (daily) and clinical (weekly) outcomes. LLM-rated activation was associated with same-day increases in positive affect and decreases in negative affect. In contrast, passive sensing features were more strongly related to weekly changes in symptoms. These distinct timescales suggest that linguistic measures may be better suited to capturing short-term emotional changes, whereas passive sensing may tap into behavioral processes that unfold over longer periods. This finding is clinically significant: it implies that daily text assessments could help clinicians monitor affective responses to activation efforts in real time, while mobility patterns could indicate whether treatment is gradually translating into symptom improvement.

Interestingly, associations between both the LLM-derived and passive sensing indicators of activation with BADS and symptom measures were significant at the within-person level, but not the between-person level. That is, when individuals showed more activation relative to *their own mean* via passive sensing or text, they also reported higher activation and lower symptoms; however, individuals who, on average, moved more or reported more engagement via EMA text were not necessarily less symptomatic. This pattern is consistent with recent research suggesting that daily mobility features, such as time spent at home or number of locations visited, are more predictive of within-person changes in depression than between-person differences [28]. Clinically, this highlights the need for personalized interventions that focus on deviations from an individual’s own baseline, rather than comparing them to normative averages that may not reflect any one individual. If replicated, our study suggests that digital measures of activation could be used to tailor treatment in real time, identifying when a patient’s activation is dropping and intervening accordingly.

### Limitations and future directions

Several limitations should be noted. First, the small sample size, specifically for the passive sensor data collected from a subset of participants, limits generalizability, and replication in larger, more diverse samples is necessary. Second, the LLM-based ratings were generated using a specific prompt and model version. Although recent work has shown that LLM-based ratings using GPT-4o are surprisingly stable and human-like [18], slight variations in prompts or model updates could influence results. Another limitation is that passive sensing features capture only certain dimensions of activation, mainly physical mobility, and may miss other important nuances. Future work should integrate additional passive data streams (e.g., phone use patterns, conversation detection, sleep onset/offset) and physiological markers to capture a broader spectrum of activation-relevant processes. Similarly, while language-based ratings provide valuable context, they depend on participant compliance and openness within a specific EMA sampling scheme. Integrating insights from NLP as applied to continuously collected text data, such as from social media and text messages, would improve the richness and ecological validity of this data stream [30].

In conclusion, this proof-of-concept study provides initial evidence that passive smartphone sensing and LLM-based language analysis can be used to measure activation in adolescents during treatment for depression, offering insights into therapeutic processes as they unfold in daily life. By validating these digital tools against self-report measures and demonstrating their links to emotional and clinical outcomes, we illustrate their potential to enhance treatment monitoring, personalize interventions, and ultimately improve outcomes for depressed youth.

## Supplementary information

SUPPLEMENTAL MATERIAL

CONSORT 2010 Flow Diagram

## Data Availability

Data are available upon reasonable request to the corresponding author (HF) and following the execution of a data use agreement.
