# Supplementary material for: Measuring activation during behavioral activation therapy: a proof-of-concept study using smartphone sensors and LLM-derived ratings in adolescents with anhedonia
Source: NPP Digit Psychiatry Neurosci. 2025 Oct 13;3:24. doi: 10.1038/s44277-025-00045-w (PMC12518126; doi:10.1038/s44277-025-00045-w)
Supplement: Supplementary file 1 — SUPPLEMENTAL MATERIAL [file 44277_2025_45_MOESM1_ESM.docx]

**Supplement**

**Section S1**

**Weekly Self-Report Measures**

***Anhedonia***

Participants reported past-week anhedonia symptoms using the SHAPS (20), a 14-item self-report questionnaire. The SHAPS includes items such as, “I would be able to enjoy my favorite meal” and “I would get pleasure from helping others.” Items were assessed using a 1 – 4 Likert scale (30), from “Strongly disagree” to “Strongly agree,” and all items were reverse-scored. Therefore, higher scores (total score range = 14 – 56) indicate more severe anhedonia. In the full sample, Cronbach’s alpha was .75.

***Depression Symptoms***

Participants reported past-week depression symptoms using the CES-D (21), a 20-item self-report measure. The CES-D includes items such as, “I felt I was just as good as other people,” and “I felt hopeful about the future.” Items were assessed using a 4-point Likert scale ranging from “rarely or none of the time (less than 1 day),” to “most or all of the time (5 - 7 days).” Therefore, higher scores indicate greater frequency of depressive symptoms. In the full sample, Cronbach’s alpha was .86.

***Activation***

Participants reported past-week activation using the BADS-SF (22), a 9-item self-report questionnaire. The BADS includes items such as, “I was an active person and accomplished the goals I set out to do,” and “I did things that were enjoyable.” The BADS-SF contains two subscales: Activation and Avoidance. Higher scores on the subscales indicate greater activation and less avoidance, respectively. In the full sample, Cronbach’s alpha for the Activation subscale was .71.

**Section S2**

**Definition of Behavioral Activation**

We define BA as a structured, brief psychotherapeutic approach that aims to (a) increase engagement in adaptive activities (which often are those associated with the experience of pleasure or mastery [self-efficacy]), (b) decrease engagement in activities that maintain depression or increase risk for depression, and (c) solve problems that limit access to reward or that maintain or increase aversive control. Treatment focuses directly on these targets or on processes that inhibit a focus on these targets (e.g., avoidance).

Dimidjian, S., Barrera Jr, M., Martell, C., Muñoz, R. F., & Lewinsohn, P. M. (2011). The origins and current status of behavioral activation treatments for depression. *Annual Review of Clinical Psychology*, *7*(1), 1-38.‏ <https://doi.org/10.1146/annurev-clinpsy-032210-104535>

**GPT-4o Prompt**

"""

Here is the definition of behavioral activation (BA):

We define BA as a structured, brief psychotherapeutic approach that aims to (a) increase engagement in adaptive activities (which often are those associated with the experience of pleasure or mastery [self-efficacy]), (b) decrease engagement in activities that maintain depression or increase risk for depression, and (c) solve problems that limit access to reward or that maintain or increase aversive control. Treatment focuses directly on these targets or on processes that inhibit a focus on these targets (e.g., avoidance).

Based on this definition, evaluate the extent to which the participant demonstrated behavioral activation (BA) based on their responses to activity-related questions.

Behavioral activation is rated on a scale from 1 to 5, where:

- 1 = Least active (passive, avoidant, reinforcing depressive behaviors).
- 2 = Low activity (minimal engagement or enjoyment).
- 3 = Moderate activity (engagement in neutral or mildly rewarding activities).
- 4 = High activity (engaged in adaptive, rewarding, or mastery-based activities).
- 5 = Very high activity (strongly reinforcing pleasure, mastery, or problem-solving).

**Guidelines for Rating:**

1. **Current activity & enjoyment:**
   - Passive activities (e.g., watching TV, lying in bed) reduce BA.
2. **Social interaction:**
   - Engaging with others increases BA, particularly through meaningful connections.
3. **Most enjoyable event:**
   - Consider the type of activity—whether it was passive (e.g., watching a show) or active (e.g., exercise, social event, creative work).
   - More engaging, mastery-oriented, or social activities increase BA ratings.
4. **Most stressful event:**
   - Consider whether the event involved problem-solving or avoidance.
   - If the participant engaged in active resolution (e.g., addressing the problem), BA remains higher.
   - If they withdrew or avoided the issue (e.g., canceling plans), BA decreases.

**Parameters:**

- participant_id (str): The unique identifier for the participant.
- time (str): The time point when the responses were recorded.
- current_activity (str): The participant’s response to what they were doing right before.
- location (str): Where the participant was doing the activity.
- interaction_type (str): Who they were interacting with (if anyone).
- most_enjoyable_event (str): The participant’s response to the most enjoyable event.
- most_enjoyable_with (str): Was anyone with them during the enjoyable event (yes/no)?
- most_enjoyable_withwhom (str): Who was with them during the enjoyable event (if anyone).
- most_stressful_event (str): The participant’s response to the most stressful event.
- most_stressful_with (str): Was anyone with them during the stressful event (yes/no)?
- most_stressful_withwhom (str): Who was with them during the stressful event (if anyone).

**Returns:**
A dictionary containing the participant’s ID, time, BA rating, and a detailed explanation of the reason for the rating.

**Example Input:**

{

"participant_id": "XXXX",

"time": "5",

"current_activity": "Watching TV",

"location": "Home",

"interaction_type": "None",

"most_enjoyable_event": "Went for a run",

"most_enjoyable_with": "No",

"most_enjoyable_withwhom": "",

"most_stressful_event": "Argued with a friend",

"most_stressful_with": "Yes",

"most_stressful_withwhom": "Friend"

}

**Example Output:**

{

"participant_id": "participant_2",

"time": "5",

"BA_rating": 2,

"reason": "The participant was engaged in passive activities such as watching TV and did not interact with anyone, pointing to high avoidance and reinforcing depressive behaviors."

}

“”"

**Note:** Ensure the explanation for the BA rating aligns with the behavioral activation definition and rating guidelines provided.
“”

See below for the EMA item wordings for each of the items used as parameters in the GPT rating (these wordings were not part of the GPT prompt, but are reported in case they are of interest):

current_activity: “Right before starting the survey, what were you doing?”

location: “Where were you doing this activity?”

interaction_type: “Who were you interacting with (in person, on phone, on computer) right before starting the survey?”

most_enjoyable_event: “Think about the most enjoyable or happy time SINCE YOU COMPLETED THE LAST SURVEY (or if this is your first survey, then the last 24 hours). Very briefly, what happened (1-2 sentences is fine)?”

most_enjoyable_with: “Was anyone with you?”

most_enjoyable_withwhom: “If yes, who was with you?”

most_stressful_event: “Think about the most stressful or negative time SINCE YOU COMPLETED THE LAST SURVEY (or if this is your first survey, then the last 24 hours). Very briefly, what happened (1-2 sentences is fine)?”

most_stressful_with: “Was anyone with you?”

most_stressful_withwhom: “If yes, who was with you?”

**Section S3**

**Passive Smartphone Measures**. The following passive sensor variables were included in the study: (1) Phone Accelerometer Score (*ActScore*): Hourly activity score of the phone accelerometer data, calculated from the standard deviation of the triaxial accelerometer data, root mean squared and scored as 1 when less than 10% (percentile), 2 when between 10% and 25%, 3 when between 25% and 50%, 4 when between 50% and 75%, 5 when between 75% and 90%; and 6 when greater than 90%. The scores are calculated minute-by-minute and then averaged to calculate the hourly values (24); (2) Daily Percent Home (*percentHome*): Percentage of time out of the available GPS time the participant spends at home which could be the Home (the most visited POI of the study) if visited on that day or otherwise, the most visited POI of the day (therefore, when the individual is travelling for several days the home location is adjusted). This parameter excludes the missing times (daily %). POIs: Maximum 80 points of interest that were detected by spatial clustering of the temporally filtered GPS coordinates visited by the participant during the entire study; (3) Distance from Home (*homDist*): If ‘Home’ is visited this parameter is 0; if not visited, this is the distance of the most visited place visited during the day (home) from Home. Basically, to detect when participant is travelling or temporarily moved to another location (daily km); (4) Daily Mobility Area (*radiusMobility*): The radius of a circle that encompasses all visited locations during the day (daily km); (5) Places Visited Daily (*DayPlaces*): Number of POIs including Home that the participant has visited during the day.

# **Section S4**

# **Missing Data Analysis**

We conducted evaluations of missing data mechanisms using Little's MCAR tests across three different analytic levels to assess the plausibility of missing completely at random (MCAR) and missing at random (MAR) assumptions in our dataset. All MCAR tests were conducted using the TestMCARNormality() function from the MissMech package in R, which implements both Hawkins' parametric test and a non-parametric alternative that is robust to normality violations.

## **Analysis 1: Weekly-Level Self-Report Measures and EMA**

- **Total cases:** 641
- **Missing data patterns:** 3

### Missing Data Patterns

| **Pattern** | **BA Ratings** | **PA** | **NA** | **BADS** | **SHAPS** | **CESD** | **N Cases** | **Percentage** |
| --- | --- | --- | --- | --- | --- | --- | --- | --- |
| 1 | + | + | + | + | + | + | 289 | 45.1% |
| 2 | + | + | + | - | - | - | 165 | 25.7% |
| 3 | - | - | - | + | + | + | 187 | 29.2% |

### MCAR Test Results

- **Non-Parametric Test p-value:** .440
- **Interpretation:** Insufficient evidence to reject MCAR at α = .05

## **Analysis 2: Weekly-Level with Self-Report, EMA, and Passive Sensing**

- **Total cases:** 283
- **Missing data patterns:** 5

### Missing Data Patterns

| **Pattern** | **BA Rating** | **PA** | **NA** | **ActScore** | **HomeDist** | **RadiusMob** | **%Home** | **NumPlaces** | **BADS** | **SHAPS** | **CESD** | **N Cases** | **Percentage** |
| --- | --- | --- | --- | --- | --- | --- | --- | --- | --- | --- | --- | --- | --- |
| 1 | + | + | + | + | + | + | + | + | + | + | + | 61 | 21.6% |
| 2 | - | - | - | + | + | + | + | + | - | - | - | 118 | 41.7% |
| 3 | + | + | + | + | + | + | + | + | - | - | - | 28 | 9.9% |
| 4 | - | - | - | + | + | + | + | + | + | + | + | 68 | 24.0% |
| 5 | + | + | + | - | - | - | - | - | - | - | - | 8 | 2.8% |

### MCAR Test Results

- **Non-Parametric Test p-value:** .269
- **Interpretation:** Insufficient evidence to reject MCAR at α = .05

## **Analysis 3: Daily-Level EMA and Passive Sensing**

- **Total cases:** 2,004 daily observations
- **Missing data patterns:** 4

### Missing Data Patterns

| **Pattern** | **BA Rating** | **ActScore** | **HomeDist** | **RadiusMob** | **%Home** | **NumPlaces** | **N Cases** | **Percentage** |
| --- | --- | --- | --- | --- | --- | --- | --- | --- |
| 1 | - | + | + | + | + | + | 1,598 | 79.7% |
| 2 | + | + | + | + | + | + | 319 | 15.9% |
| 3 | - | + | - | - | - | - | 57 | 2.8% |
| 4 | + | - | - | - | - | - | 30 | 1.5% |

### MCAR Test Results

- **Non-Parametric Test p-value:** < .000
- **Interpretation:** MCAR hypothesis rejected at α = .05

## **MAR Plausibility Analysis**

To further examine whether missingness was systematically related to other observed variables at the daily level, we followed a common approach of running a series of multilevel models (MLMs) in which missingness on each study variable was treated as the outcome and the remaining variables were entered as predictors. These models included both passive data and GPT-based behavioral activation ratings. While the analysis converged for the GPT-based ratings, the model did not converge for the passive data. Probably due to the fact that the passive variables shared the same missing-data pattern (e.g., when the device was off or data upload failed, all passive streams were missing simultaneously). Because of this redundancy, the model lacked sufficient variability to estimate parameters, leading to non-convergence. Therefore, we report results only for the GPT-based analysis. This should be viewed as a structural limitation of our missingness evaluation for passive data rather than evidence of a MAR violation.

### Logistic Regression: GPT-based ratings Missingness Predicted by Observed Passive Variables

#### Model Results

| **Predictor** | **Coefficient** | **SE** | **z-value** | **p-value** | **Significance** |
| --- | --- | --- | --- | --- | --- |
| (Intercept) | 3.048 | 0.637 | 4.783 | < 0.001 | *** |
| ActScore | -0.096 | 0.130 | -0.739 | 0.460 |  |
| homDist | -0.0001 | 0.00005 | -2.366 | 0.018 | * |
| radiusMobility | 0.0008 | 0.0008 | 1.001 | 0.317 |  |
| percentHome | -0.012 | 0.005 | -2.332 | 0.020 | * |
| numPlaces | -0.042 | 0.034 | -1.254 | 0.210 |  |

#### Model Statistics

- **N observations:** 1,917
- **Interpretation:** some support for MAR

**Table S1**

*Demographic and Clinical Characteristics of the Sample*

| \| **Sample Characteristics (*N* = 38)** \| \| --- \| | | |
| --- | --- | --- | --- |
|  | ***n*** | **%** |
| **Biological Sex** |  |  |
| Female | 26 | 68.4 |
| Male | 12 | 31.6 |
|  |  |  |
| **Race** |  |  |
| American Indian or Alaska Native | 0 | 0.0 |
| Asian | 3 | 7.9 |
| Black or African American | 1 | 2.6 |
| Native Hawaiian or Pacific Islander | 0 | 0.0 |
| White | 30 | 78.9 |
| Multiracial | 4 | 10.5 |
|  |  |  |
| **Ethnicity** |  |  |
| Hispanic or Latino | 1 | 2.6 |
| Not Hispanic or Latino | 37 | 97.4 |
|  |  |  |
| **Baseline Diagnoses (DSM-V)** |  |  |
| Major Depressive Episode | 23 | 60.5 |
| Generalized Anxiety Disorder | 8 | 21.1 |
| Social Anxiety Disorder | 5 | 13.2 |
| Panic Disorder | 1 | 2.6 |
| Specific Phobia | 1 | 2.6 |
| Attention-Deficit / Hyperactivity Disorder | 3 | 7.9 |
| Oppositional Defiant Disorder | 2 | 5.3 |
| Post-Traumatic Stress Disorder | 1 | 2.6 |
|  |  |  |
| **Medication** |  |  |
| Psychotropic Medication | 7 | 18.4 |
|  |  |  |
|  | **Mean (Range)** | ***SD*** |
| **Age (years)** | 15.8 (13 - 18) | 1.9 |
| **Family Income (dollars)** | 128,366.70  (20,000 - 350,000) | 69,382.75 |

| **Table S2** | |
| --- | --- |
| *EMA Compliance During Treatment* | |
| Week | Mean Compliance (%) |
| 1-2 | 62 |
| 3-4 | 58 |
| 5-6 | 51 |
| 7-8 | 53 |
| 9-10 | 53 |
| 11-12 | 52 |

**Table S3**

*Descriptive Statistics for Daily Assessments of Passive Sensing Features and GPT-Derived Activation*

| **Variable** | **N** | **Mean** | **SD** | **Min** | **Max** | **Skewness** | **Kurtosis** |
| --- | --- | --- | --- | --- | --- | --- | --- |
| ActScore | 1978 | 3.60 | 0.50 | 1.1 | 5.61 | 0.12 | 4.05 |
| homDist | 1919 | 311.80 | 1048.49 | 0.0 | 12716.66 | 5.14 | 45.59 |
| radiusMobility | 1919 | 25.82 | 194.00 | 0.0 | 4191.80 | 15.91 | 291.00 |
| percentHome | 1919 | 76.72 | 16.70 | 21.0 | 100.00 | -0.34 | 2.34 |
| DayPlaces | 1919 | 3.86 | 2.34 | 1.0 | 22.00 | 1.14 | 5.81 |
| GPT-derived activation | 1909 | 2.89 | 0.99 | 1 | 5 | -0.56 | 2.54 |

*Note. ActScore* – Daily phone accelerometer activity score. *homDist* – Maximum distance (in km) from home traveled that day. *radiusMobility* – Radius (in km) covering all places visited in a day. *percentHome* – Percentage of time spent at home each day. *DayPlaces* – Number of distinct locations visited each day.

**Table S4**

*Multilevel Model Results of Passive Sensor Variables Predicting Daily Negative and Positive Affect, Controlling for Prior-Day Affect*

|  |  | **Negative Affect** | | |  | **Positive Affect** | | | |
| --- | --- | --- | --- | --- | --- | --- | --- | --- | --- |
| **Term** | **N** | **Estimate (SE)** | **Statistic** | ***p* value** | **95% CI** | **Estimate (SE)** | **Statistic** | ***p* value** | **95% CI** |
| (Intercept) | 208 | -18.43 (12.83) | -1.44 | 0.180 | [-46.82, 9.95] | 16.86 (7.16) | 2.36 | .046 | [0.388, 33.34] |
| Lag outcome _person-centered_ | 208 | 0.048 (0.070) | 0.684 | 0.495 | [-0.090, 0.185] | 0.359 (0.067) | 5.37 | <.001 | [0.227, 0.491] |
| ActScore _person-centered_ | 208 | 0.027 (0.066) | 0.415 | 0.678 | [-0.102, 0.157] | 0.094 (0.077) | 1.22 | .223 | [-0.058, 0.245] |
| ActScore _mean_ | 208 | 5.46 (3.56) | 1.53 | 0.154 | [-2.41, 13.34] | -4.45 (1.99) | -2.24 | .055 | [-9.02, 0.126] |
| (Intercept) | 199 | -5.56 (2.69) | -2.07 | 0.065 | [-11.54, 0.423] | 0.351 (2.28) | 0.154 | .881 | [-4.74, 5.44] |
| Lag outcome _person-centered_ | 199 | 0.043 (0.071) | 0.607 | 0.545 | [-0.097, 0.184] | 0.330 (0.071) | 4.63 | <.001 | [0.189, 0.470] |
| HourPlaces _person-centered_ | 199 | 0.200 (0.295) | 0.678 | 0.499 | [-0.382, 0.783] | 0.449 (2.03) | 0.221 | .830 | [-4.08, 4.98] |
| HourPlaces _mean_ | 199 | **6.07 (2.40)** | **2.53** | **0.029** | **[0.741, 11.39]** | 0.330 (0.071) | 4.63 | <.001 | [0.189, 0.470] |
| (Intercept) | 207 | 1.30 (0.246) | 5.28 | 0.000 | [0.756, 1.84] | 0.875 (0.161) | 5.44 | <.001 | [0.517, 1.23] |
| Lag outcome _person-centered_ | 207 | 0.052 (0.069) | 0.758 | 0.449 | [-0.084, 0.189] | 0.363 (0.067) | 5.45 | <.001 | [0.232, 0.494] |
| homDist _person-centered_ | 207 | 0.000 (0.000) | 0.155 | 0.877 | [-0.000, 0.001] | 0.000 (0.000) | 1.44 | .151 | [-0.000, 0.001] |
| homDist _mean_ | 207 | -0.000 (0.000) | -0.495 | 0.630 | [-0.001, 0.001] | -0.000 (0.000) | -0.387 | .705 | [-0.001, 0.001] |
| (Intercept) | 207 | 1.69 (0.375) | 4.50 | 0.001 | [0.860, 2.51] | 0.643 (0.258) | 2.49 | .032 | [0.067, 1.22] |
| Lag outcome _person-centered_ | 207 | 0.052 (0.069) | 0.752 | 0.453 | [-0.085, 0.189] | 0.361 (0.066) | 5.46 | <.001 | [0.230, 0.491] |
| radiusMobility _person-centered_ | 207 | 0.000 (0.001) | 0.357 | 0.722 | [-0.001, 0.001] | **0.001 (0.001)** | **2.09** | **.038** | **[0.000, 0.003]** |
| radiusMobility _mean_ | 207 | -0.020 (0.014) | -1.41 | 0.187 | [-0.050, 0.011] | 0.011 (0.010) | 1.10 | .295 | [-0.011, 0.032] |
| (Intercept) | 207 | 0.945 (2.58) | 0.367 | 0.721 | [-4.72, 6.61] | 3.20 (1.53) | 2.09 | .062 | [-0.195, 6.60] |
| Lag outcome _person-centered_ | 207 | 0.051 (0.069) | 0.741 | 0.460 | [-0.085, 0.188] | 0.368 (0.067) | 5.50 | <.001 | [0.236, 0.500] |
| percentHome _person-centered_ | 207 | -0.002 (0.002) | -0.673 | 0.502 | [-0.006, 0.003] | -0.000 (0.003) | -0.127 | .899 | [-0.006, 0.005] |
| percentHome _mean_ | 207 | 0.004 (0.033) | 0.119 | 0.908 | [-0.069, 0.076] | -0.030 (0.020) | -1.53 | .155 | [-0.074, 0.013] |
| (Intercept) | 207 | -0.309 (0.754) | -0.410 | 0.690 | [-1.98, 1.36] | 0.701 (0.608) | 1.15 | .273 | [-0.635, 2.04] |
| Lag outcome _person-centered_ | 207 | 0.051 (0.069) | 0.731 | 0.466 | [-0.086, 0.188] | 0.363 (0.067) | 5.43 | <.001 | [0.231, 0.495] |
| DayPlaces _person-centered_ | 207 | 0.007 (0.018) | 0.366 | 0.715 | [-0.029, 0.042] | 0.019 (0.021) | 0.926 | .356 | [-0.022, 0.061] |
| DayPlaces _mean_ | 207 | 0.389 (0.183) | 2.13 | 0.058 | [-0.016, 0.795] | 0.041 (0.147) | 0.281 | .784 | [-0.283, 0.365] |

*Note. ActScore* – Daily phone accelerometer activity score. *homDist* – Maximum distance (in km) from home traveled that day. *radiusMobility* – Radius (in km) covering all places visited in a day. *percentHome* – Percentage of time spent at home each day. *DayPlaces* – Number of distinct locations visited each day. Variables labeled *person-centered* reflect day-to-day fluctuations within individuals. Variables labeled *mean* reflect each participant’s average across the study period.

**Table S5**

Multilevel Model Results of Passive Sensing Variables Predicting Weekly Self-Reported Activation and Avoidance Subscales

|  |  | **Activation** | | | | **Avoidance** | | | |
| --- | --- | --- | --- | --- | --- | --- | --- | --- | --- |
| **Term** | **N** | **Estimate (SE)** | **Statistic** | ***p* value** | **95% CI** | **Estimate (SE)** | **Statistic** | ***p* value** | **95% CI** |
| (Intercept) | 147 | -51.13 (48.44) | -1.06 | .315 | [-158.79, 56.52] | -25.18 (26.20) | -0.961 | .359 | [-83.35, 32.99] |
| ActScore _person-centered_ | 147 | -3.11 (4.25) | -0.731 | .466 | [-11.53, 5.30] | -0.689 (2.27) | -0.304 | .762 | [-5.18, 3.80] |
| ActScore _mean_ | 147 | 18.95 (13.82) | 1.37 | .200 | [-11.77, 49.68] | 9.76 (7.48) | 1.31 | .220 | [-6.84, 26.36] |
| (Intercept) | 147 | 14.47 (1.35) | 10.70 | .000 | [11.46, 17.48] | 8.77 (0.800) | 10.95 | <.001 | [6.99, 10.54] |
| homDist _person-centered_ | 147 | 0.001 (0.002) | 0.401 | .689 | [-0.004, 0.005] | 0.000 (0.001) | 0.296 | .767 | [-0.002, 0.003] |
| homDist _mean_ | 147 | 0.003 (0.002) | 1.77 | .108 | [-0.001, 0.007] | 0.001 (0.001) | 0.893 | .393 | [-0.001, 0.003] |
| (Intercept) | 147 | 12.38 (2.65) | 4.67 | .001 | [6.47, 18.30] | 6.55 (1.23) | 5.32 | <.001 | [3.80, 9.31] |
| radiusMobility _person-centered_ | 147 | 0.038 (0.022) | 1.72 | .087 | [-0.006, 0.081] | 0.012 (0.012) | 1.05 | .295 | [-0.011, 0.036] |
| radiusMobility _mean_ | 147 | 0.215 (0.169) | 1.27 | .233 | [-0.163, 0.593] | **0.183 (0.079)** | **2.33** | **.043** | **[0.007, 0.359]** |
| (Intercept) | 147 | 45.03 (16.41) | 2.74 | .021 | [8.41, 81.65] | 33.00 (6.64) | 4.97 | .001 | [18.13, 47.87] |
| percentHome _person-centered_ | 147 | **-0.178 (0.058)** | **-3.06** | **.003** | **[-0.294, -0.063]** | **-0.068 (0.032)** | **-2.16** | **.033** | **[-0.131, -0.006]** |
| percentHome _mean_ | 147 | -0.370 (0.203) | -1.82 | .099 | [-0.824, 0.083] | **-0.298 (0.082)** | **-3.62** | **.005** | **[-0.482, -0.114]** |
| (Intercept) | 147 | 6.27 (6.49) | 0.966 | .358 | [-8.25, 20.78] | 3.51 (3.36) | 1.04 | .322 | [-4.01, 11.02] |
| DayPlaces _person-centered_ | 147 | **1.60 (0.465)** | **3.45** | **.001** | **[0.684, 2.52]** | **0.600 (0.254)** | **2.37** | **.019** | **[0.098, 1.10]** |
| DayPlaces _mean_ | 147 | 2.44 (1.73) | 1.41 | .190 | [-1.43, 6.30] | 1.49 (0.895) | 1.67 | .127 | [-0.508, 3.49] |

*Note. ActScore* – Daily phone accelerometer activity score. *homDist* – Maximum distance (in km) from home traveled that day. *radiusMobility* – Radius (in km) covering all places visited in a day. *percentHome* – Percentage of time spent at home each day. *DayPlaces* – Number of distinct locations visited each day. Variables labeled *person-centered* reflect day-to-day fluctuations within individuals. Variables labeled *mean* reflect each participant’s average across the study period.

**Table S6**

Multilevel Model Results of GPT-Derived Activation Ratings Predicting Weekly Self-Reported Activation and Avoidance Subscales

| **Outcome** | **Term** | **N** | **Estimate (SE)** | **Statistic** | ***p* value** | **95% CI** |
| --- | --- | --- | --- | --- | --- | --- |
| Activation  scale | (Intercept) | 289 | 7.08 (6.52) | 1.09 | .285 | [-6.16, 20.32] |
|  | GPT rating _person-centered_ | **289** | **1.62 (0.555)** | **2.92** | **.004** | **[0.530, 2.72]** |
|  | GPT rating _mean_ | 289 | 2.81 (2.20) | 1.28 | .210 | [-1.66, 7.29] |
| Avoidance  scale | (Intercept) | 289 | 6.64 (3.34) | 1.99 | .055 | [-0.156, 13.43] |
|  | GPT rating _person-centered_ | 289 | 0.428 (0.299) | 1.43 | .154 | [-0.162, 1.02] |
|  | GPT rating _mean_ | 289 | 0.965 (1.13) | 0.854 | .399 | [-1.33, 3.26] |

*Note.* Variables labeled *person-centered* reflect day-to-day fluctuations within individuals. Variables labeled *mean* reflect each participant’s average across the study period.

|  | **Convergent** | | | **Criterion** | | | |
| --- | --- | --- | --- | --- | --- | --- | --- |
| Passive Sensing Variable | GPT-rated BA  (Daily) | BADS  (Weekly) | Negative Affect  (Daily) | | Positive Affect  (Daily) | SHAPS  (Weekly) | CES-D  (Weekly) |
| Activity Score | + (WP) |  |  | |  |  |  |
| Home Distance |  |  |  | |  |  |  |
| Radius of Mobility |  |  |  | | + (WP) |  |  |
| Percent Time at Home | - (WP) | - (BP,WP) |  | |  | + (WP) | + (WP) |
| Number of Places Visited Daily | + (WP) | + (WP) |  | |  | - (WP) | - (WP) |

**Table S7**

*Associations Between Passive Sensing Variables, GPT-Rated Activation, and Self-Reports*

*Note*. WP: Within-Person, BP: Between-person, BADS: Behavioral Activation for Depression Scale

**Table S8**

*Examples of participants free text entry and GPT-4o ratings and reasoning*

| **Current activity** | **Enjoying event** | **Stressful event** |  |  |
| --- | --- | --- | --- | --- |
| At the bus stop taking the subway | Nothing enjoyable | I weigh myself and think that I'm too fat | 1 | The participant did not report any enjoyable activities, showing no engagement in adaptive or rewarding behaviors. The current activity of taking the subway is neutral and involves no social interaction. Additionally, the most stressful event involves negative self-evaluation without any indication of problem-solving or active coping, suggesting avoidance and reinforcing depressive thoughts. |
| Sleeping in the car | Movie with family | Realizing how much work I have due Monday | 1 | The participant was engaged in a passive activity (sleeping in the car) and did not interact with anyone, indicating avoidance. The most enjoyable event was a passive activity (watching a movie) despite being social, limiting active engagement or mastery. The most stressful event involved avoidance of problem-solving, as there is no indication they actively addressed the work due. These factors collectively suggest a low level of behavioral activation. |
| preparing to watch infinity war | I listened to Florence and the machine | my mom was watching the show i was watching over my shoulder and it pissed me off. i didn't do or say anything though | 2 | The participant was preparing to watch a passive activity (watching a movie) and was interacting with their mom in the process, which indicates low activity and minimal social engagement. The most enjoyable event, listening to music, was also a relatively passive activity done alone. During the most stressful event, the participant chose not to address the frustration with their mom, indicating avoidance behavior. Overall, these factors point to low levels of behavioral activation. |
| Taking photos of clothes at home with my friend | Sleeping | Checking for job application progress | 3 | The participant was engaged in a mildly active task of taking photos of clothes with a friend, showing some engagement and social interaction. However, the most enjoyable event was passive (sleeping), indicating limited reinforcement of pleasure through active engagement. The stressful event involved checking job application progress, which reflects engagement in an aversive situation but not avoidance. The combination of mild activity, passive enjoyment, and non-avoidant stressful engagement suggests a moderate level of behavioral activation. |
| Stretching in my bedroom | I went to a dance lesson | my dad wouldn't let me use his credit card to buy something that I need online so I have to wait for my brother to get home to use the other one | 4 | The participant engaged in adaptive and rewarding activities, such as stretching and attending a dance lesson, which increases BA. Although the current activity was done alone, attending the dance lesson involved social interaction with a teacher, contributing to meaningful engagement. The issue of needing a credit card could be seen as problematic, but the participant planned to resolve it by waiting for their brother, demonstrating some problem-solving approach rather than avoidance. |
| Returning home from an orchestra concert. | Putting on an event for the younger kids in my community. With Some of my friends and instructors. | Sitting through a school assembly where most students were being thoroughly disrespectful toward the presenters and each other. With my classmates and a few teachers. | 5 | The participant demonstrated very high behavioral activation by engaging in an active, social, and mastery-oriented event, namely organizing an event, which involved interaction with friends and instructors. The participant also attended an orchestra concert with family, which is an adaptive and rewarding activity. Even though the most stressful event involved being in an uncomfortable situation, the participant's overall engagement in meaningful and social activities outweighs this, reinforcing pleasure and mastery. |

**Figure S1**

*Repeated Measures Correlations Between Passive Sensor Variables and GPT-Derived Activation*


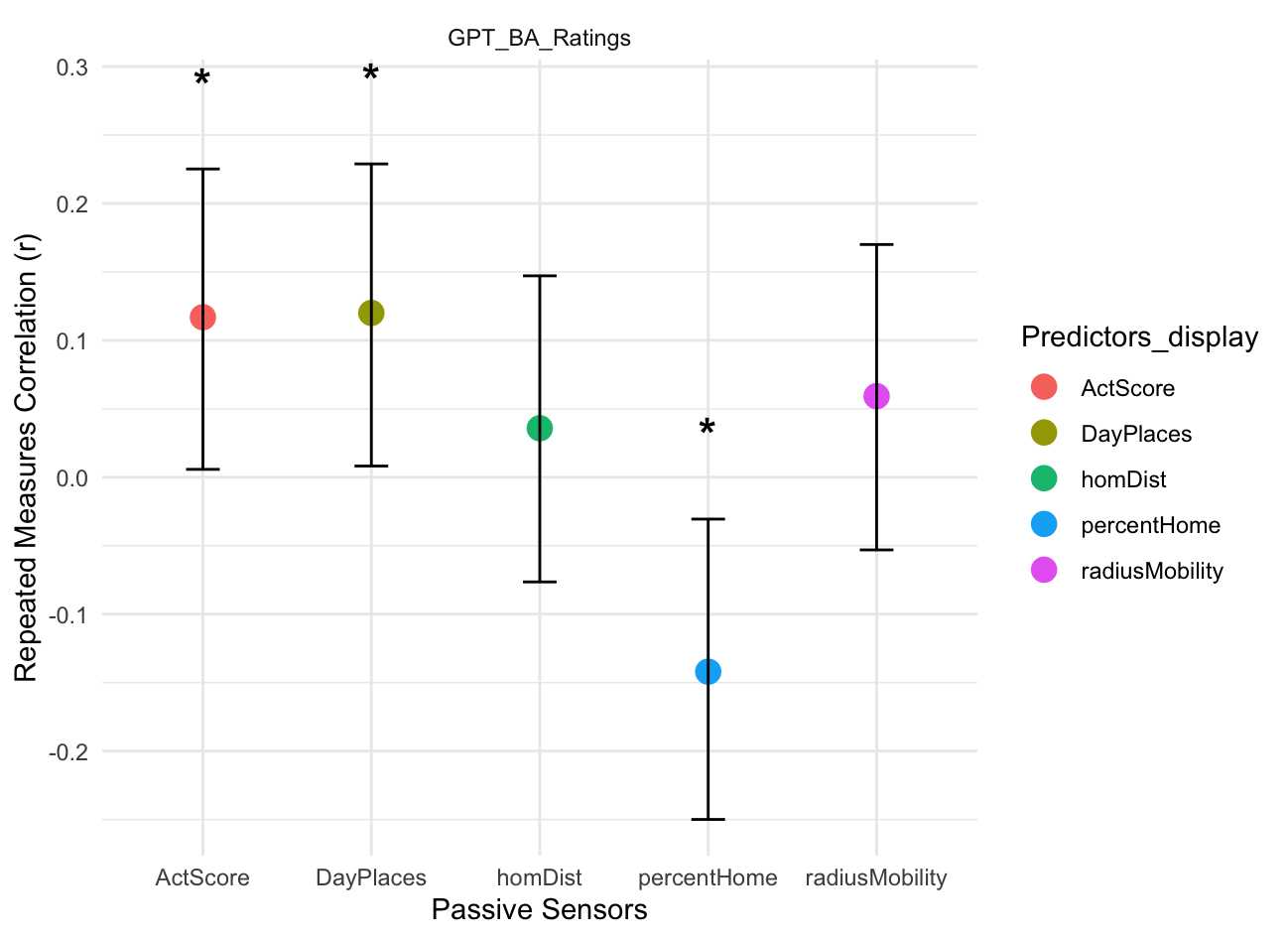


**Figure S2**

*Change in GPT-Derived Activation Ratings and Self-Reported Activation Over Time by Participant*


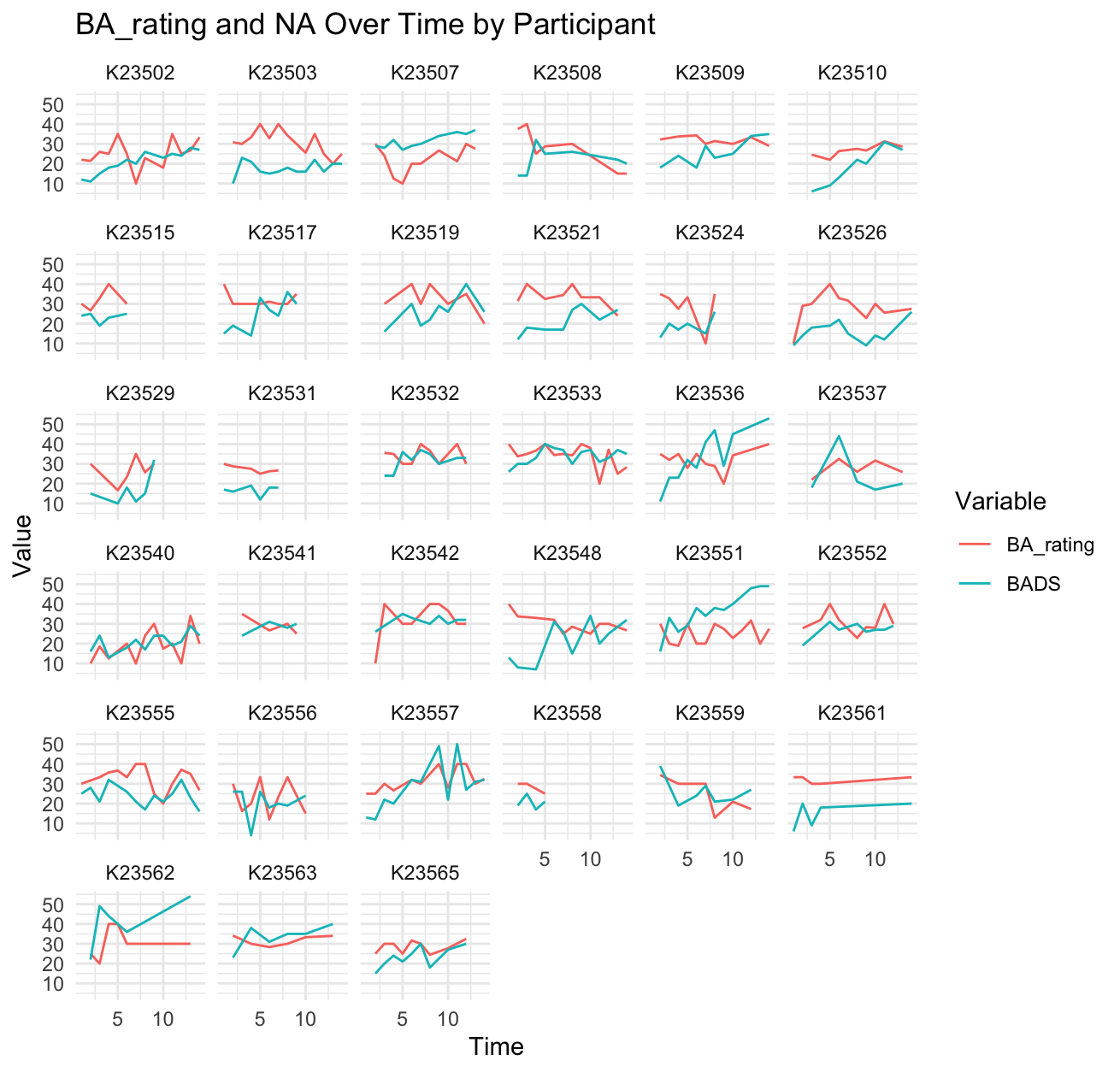


Participant 1

Participant 2

Participant 3

Participant 4

Participant 5

Participant 6

Participant 7

Participant 8

Participant 9

Participant 10

Participant 11

Participant 12

Participant 12

Participant 13

Participant 14

Participant 15

Participant 16

Participant 17

Participant 18

Participant 19

Participant 20

Participant 21

Participant 22

Participant 23

Participant 24

Participant 25

Participant 26

Participant 27

Participant 28

Participant 29

Participant 30

Participant 31

Participant 32

**Figure S3**

*Repeated Measures Correlations of Passive Sensor Variables Predicting Self-Reported Activation (BADS): (a) Total Scale, (b) Activation and Avoidance Subscales*

a.


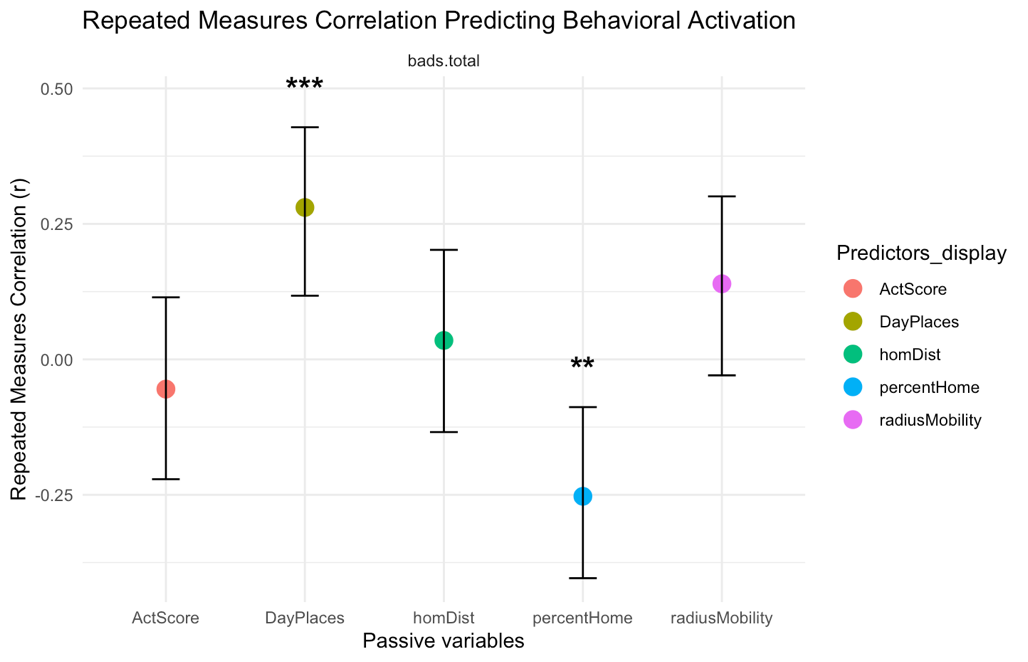


b.

Avoidance scale

Activation scale


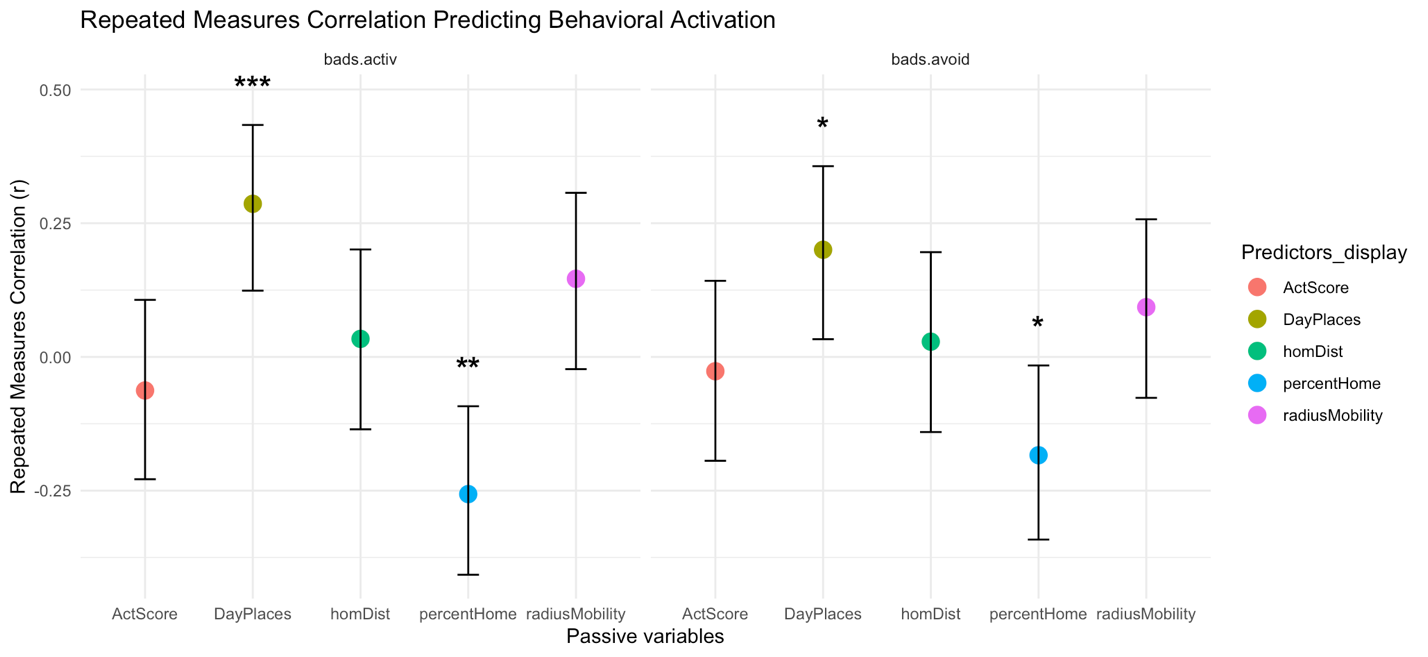


**Figure S4**

*Repeated Measures Correlations of Passive Sensor Variables Predicting Daily Positive and Negative Affect*

Positive Affect

Negative Affect

*
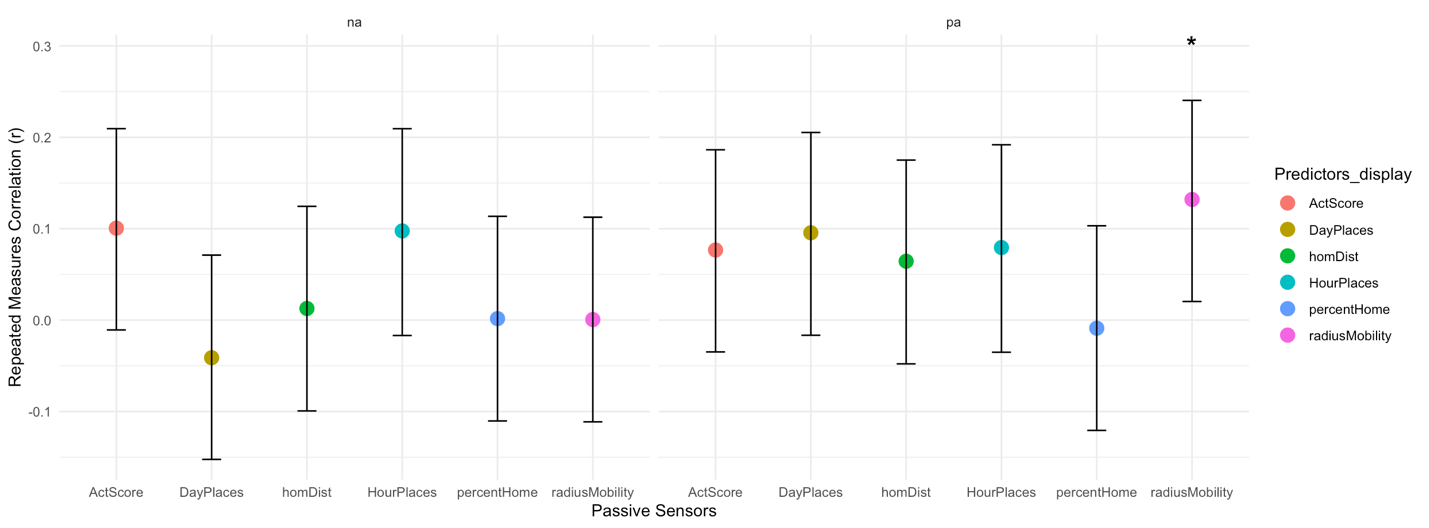
*

**Figure S5**

*Repeated Measure Correlations of Passive Sensors Predicting Weekly Anhedonia (SHAPS) and Depression (CES-D)*


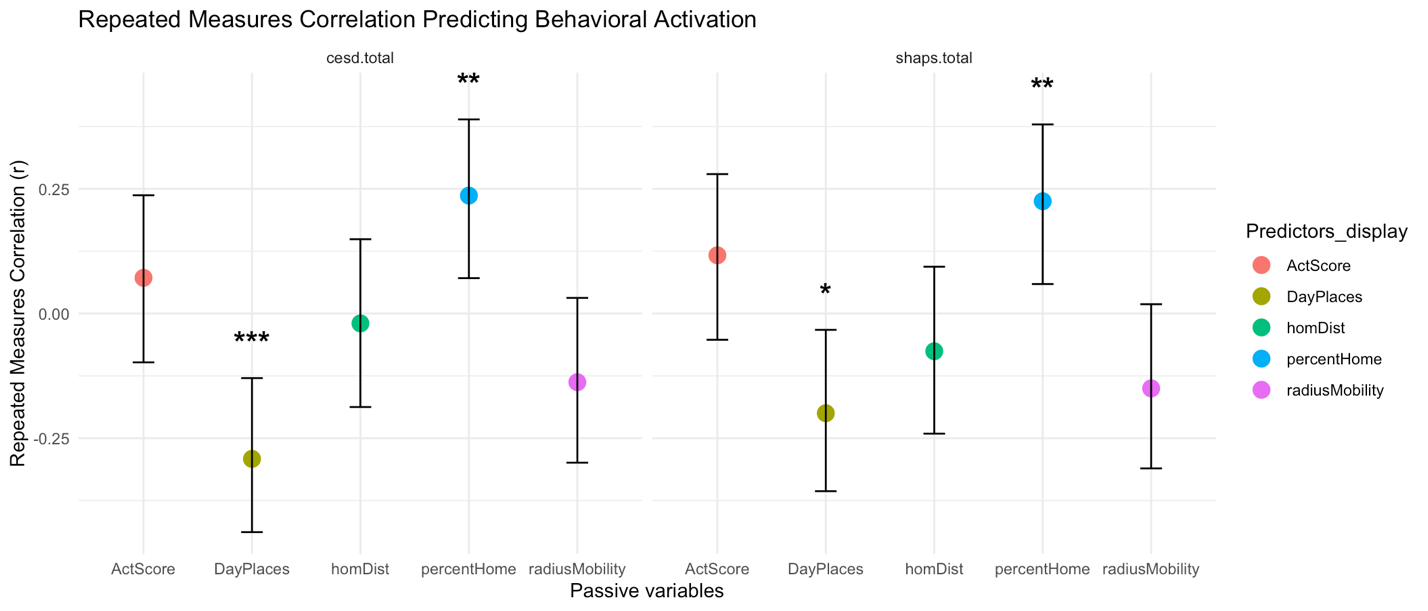


Anhedonia (SHAPS)

Depression (CES-D)
