# Supplementary figures and images for: Measuring activation during behavioral activation therapy: a proof-of-concept study using smartphone sensors and LLM-derived ratings in adolescents with anhedonia
Source: NPP Digit Psychiatry Neurosci. 2025 Oct 13;3:24. doi: 10.1038/s44277-025-00045-w (PMC12518126; doi:10.1038/s44277-025-00045-w)

## CONSORT 2010 Flow Diagram

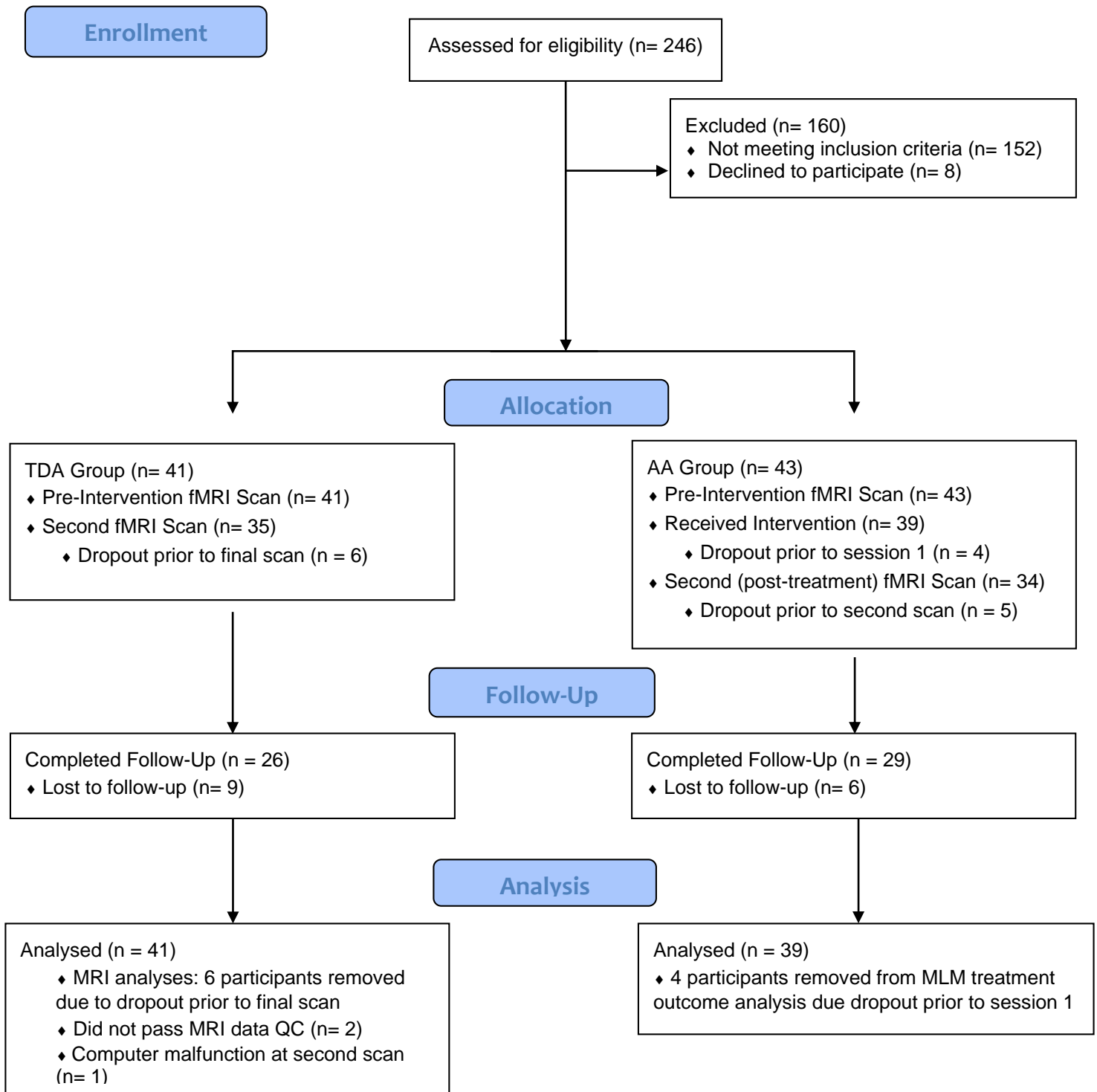

Supplement: Supplementary file 2 — CONSORT 2010 Flow Diagram [file 44277_2025_45_MOESM2_ESM.pdf]
